# Sorafenib inhibits cell growth but fails to enhance radio- and chemosensitivity of glioblastoma cell lines

**DOI:** 10.18632/oncotarget.11328

**Published:** 2016-08-17

**Authors:** Matthias Riedel, Nina Struve, Justus Müller-Goebel, Sabrina Köcher, Cordula Petersen, Ekkehard Dikomey, Kai Rothkamm, Malte Kriegs

**Affiliations:** ^1^ Laboratory of Radiobiology & Experimental Radiooncology, University Medical Center Hamburg – Eppendorf, Hubertus Wald Tumorzentrum – University Cancer Center Hamburg, 20246 Hamburg, Germany

**Keywords:** glioblastoma, sorafenib, X-irradiation, radiochemosensitivity, temozolomide

## Abstract

**Background:**

Glioblastomas (GBM) are the most common malignant type of primary brain tumor. GBM are intensively treated with surgery and combined radiochemotherapy using X-irradiation and temozolomide (TMZ) but they are still associated with an extremely poor prognosis, urging for the development of new treatment strategies. To improve the outcome of GBM patients, the small molecule multi-kinase inhibitor sorafenib has moved into focus of recent research. Sorafenib has already been shown to enhance the radio- and radiochemosensitivity of other tumor entities. Whether sorafenib is also able to sensitize GBM cells to radio- and chemotherapy is still an unsolved question which we have addressed in this study.

**Methods:**

The effect of sorafenib on signaling, proliferation, radiosensitivity, chemosensitivity and radiochemosensitivity was analyzed in six glioblastoma cell lines using Western blot, proliferation- and colony formation assays.

**Results:**

In half of the cell lines sorafenib clearly inhibited MAPK signaling. We also observed a strong blockage of proliferation, which was, however, not associated with MAPK pathway inhibition. Sorafenib had only minor effects on cell survival when administered alone. Most importantly, sorafenib treatment failed to enhance GBM cell killing by irradiation, TMZ or combined treatment, and instead rather caused resistance in some cell lines.

**Conclusion:**

Our data suggest that sorafenib treatment may not improve the efficacy of radiochemotherapy in GBM.

## INTRODUCTION

Glioblastoma multiforme (GBM), a high-grade glioma (WHO grade IV) is the most common and lethal primary malignant brain tumor in adults, with a median survival of only 16 months. Despite current intensive therapy regimes including surgery, radiotherapy (RT) and temozolomide (TMZ)-based adjuvant chemotherapy (CT), disease progression occurs in almost all patients [1, 2]. Therefore, the improvement of therapy for GBM patients is in the focus of recent research, which also includes targeted therapeutics to inhibit cellular signaling pathways [3, 4].

This includes the promising approach of using the multi-kinase inhibitor sorafenib. Sorafenib has been shown not only to block the members of the MAPK pathways Raf-1 and p38 but also receptor tyrosine kinases like VEGFR, cKit or PDGFR [5] and it is already approved for the treatment of various tumor entities [6–8]. For GBM cells sorafenib has been shown to induce apoptosis, to deplete tumor initiating cells and to reduce proliferation in cell culture and in xenograft models [9–11]. Despite these promising results sorafenib showed only very limited effects as a mono-therapeutic drug, in combination with TMZ or other targeted therapeutics such as erlotinib in clinical studies with patients having progressive or recurrent diseases [12–16]. However, for other entities we and others have already reported that sorafenib induces cellular radiosensitization, arguing for a combination of radiotherapy and sorafenib to improve the treatment of radioresistant tumors [17–21]. For GBM cells so far only two studies exist which tested the combination of sorafenib and X-irradiation by determining the number of viable cells or by using the MTT assay respectively [22, 23]. Despite some promising results, these data certainly do not answer the question of cellular radiosensitization by sorafenib.

Because of the importance of sorafenib for current targeted therapy approaches and the lack of solid data on the effects of sorafenib on X-irradiation and TMZ in GBM we investigated in this study the potential of sorafenib to radiosensitize and chemosensitize GBM cells. This study was performed using six individual GBM cell lines with differences in the p53 status, because the p53 status is known to be important for cell survival. Furthermore, we only used O6-methylguanine-methyltransferase (MGMT) negative cells since the TMZ sensitivity is known to depend strongly on MGMT status [24].

## RESULTS

To test if sorafenib is a potential therapeutic drug to improve radio-chemotherapy of GBM we wanted to analyze the influence of sorafenib on cellular radio- and chemosensitivity in various GBM cells lines. To this end, we chose the colony-forming assay, because this assay is able to directly measure the ability of tumor cells for self-renewal (clonogenicity). This is of special importance since effects on proliferation or metabolism might not truly reflect cell inactivation but could be also be caused by prolonged growth arrest. Withdrawal of the inhibitor, re-stimulating events or extended culture times might lead to a restart in growth of solely arrested but not truly inactivated cells.

### Impact of sorafenib on proliferation, clonogenicity and MAPK signaling

**Figure 1:**
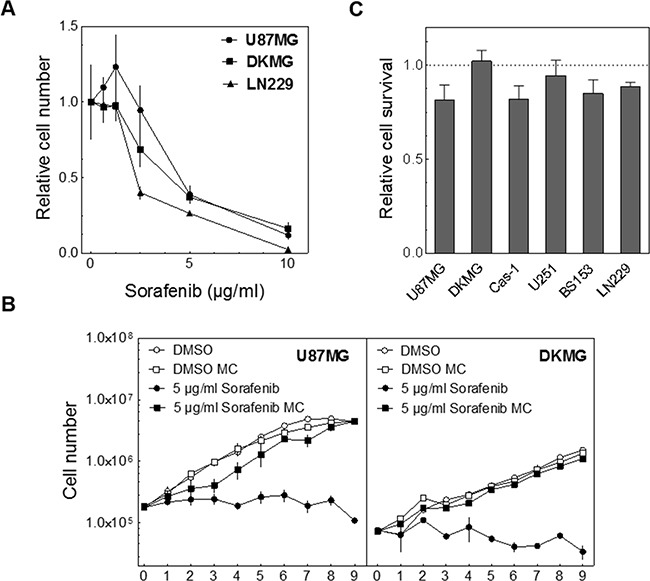
Effect of sorafenib on proliferation & clonogenicity **A.** To determine the effect of sorafenib on proliferation U87MG, DKMG and LN229 cells were incubated with different concentrations of sorafenib for 3 days as indicated. The number of cells measured in treated cultures was divided by the number of cells determined for untreated cultures, both counted after 3 days of incubation. The relative cell number is depicted. **B.** Proliferation of U87MG and DKMG cells in the presence of sorafenib (n=2). Twenty-four hours after seeding the cells were treated with 5 μg/ml sorafenib either for 24 h (media change, MC) or for up to 9 d. **C.** Relative cytotoxicity as determined by colony forming assay. Cells were treated with 5 μg/ml sorafenib for 24 h and cultivated for 10-25 days to allow for colony formation.

Because the colony forming assay can be influenced by the proliferation rate we first investigated the effect of sorafenib on cell proliferation. In these experiments we observed a strong decrease in the proliferation rate for all cell lines in a concentration-dependent manner (Figure 1A).

Because this block in proliferation might reduce clonogenicity and thereby mimic cell inactivation we tested if the inhibitory effect of sorafenib persists. For these and further experiments we chose a concentration of 5 μg/ml sorafenib tosylate which matches 7.8 μM and therefore approximately reflects the serum level in patients [12, 13, 25]. When the cells were exposed continuously sorafenib caused a complete block of proliferation for up to 9 days which was statistically significant from the 2^nd^ day on (Figure 1B). In contrast, when sorafenib was removed after 24 h the cells were able to proliferate again. Under these conditions only moderate cytotoxic effects were observable using the colony forming assays (Figure 1C, pre-plating). Therefore the medium was changed 24 h after the treatment in the following assays.

**Figure 2:**
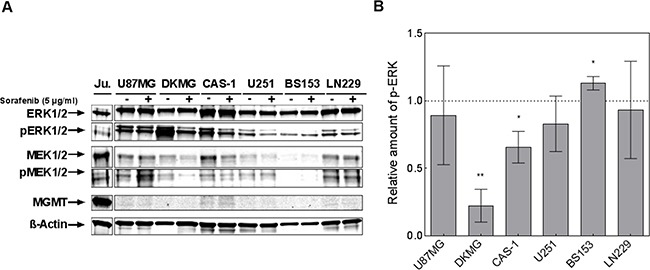
Effect of sorafenib on MAPK signaling **A.** Phosphorylation of ERK1/2 (T202/Y204) and MEK1/2 (S217/S221) was determined by Western blot analysis using phosphospecific antibodies. Cells were treated with 5 μg/ml sorafenib for 2 h. The detection of total ERK, MEK and β-actin served as controls. MGMT was detected using MGMT-specific antibodies while lysates from Jurkat cells were used as a positive control. **B.** Quantification of ERK1/2 phosphorylation after sorafenib treatment. Corrected pERK1/2 levels (pERK/ERK) of sorafenib-treated samples were normalized to the corrected pERK1/2 levels of untreated samples. Depicted are the results of three independent Western blots.

Next, the MAPK signaling pathway was analyzed, since Raf-1 is a main target of sorafenib [5]. We observed reduced MEK1/2 and ERK1/2-phosphorylation in at least three cell lines using Western blot analysis (Figure 2A), with significant inhibition of ERK being detectable for DKMG (p=0.003) and Cas-1 (p=0.043) cells (Figure 2B). For DKMG cells, which displayed the most impressive inhibition, we also analyzed the phosphorylation of Akt, STAT3 and VEGFR. We detected no reduction in protein phosphorylation except for Akt, albeit only when using higher concentrations of sorafenib (Supplementary Figure S1). Interestingly neither the cytotoxic effect of sorafenib nor the inhibition of proliferation correlated with the inhibition of MAPK-signaling.

### Impact of sorafenib on cellular radiosensitivity

**Figure 3:**
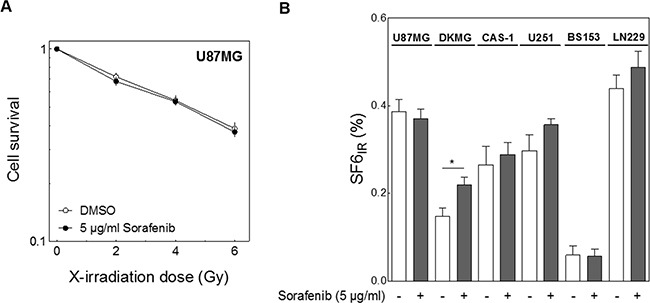
Effect of sorafenib on cellular radiosensitivity Cells were treated with 5 μg/ml sorefenib for 2 h before irradiation with 0, 2, 4 and 6 Gy. Cell survival was assessed by colony forming assay. **A.** Relative cell survival of U87MG cells. **B.** Relative surviving fraction of all six GBM cell lines after 6 Gy (SF6_IR_).

The central aim of this study was to determine whether sorafenib enhances radiosensitivity of GBM cells. Using the colony-forming assay we observed no increase in cellular radiosensitivity following sorafenib treatment in U87MG cells (Figure 3A) or in any of the other GBM cell lines as represented by the survival fraction at 6 Gy in Figure 3B. Remarkably, sorafenib might induce some radioresistance which, however, was significant only in DKMG cells (p=0.047). As reported previously [26] a huge variation in cellular radiosensitivity was seen for the six tested GBM cell lines, with LN229 cells being the most resistant and BS153 the most sensitive.

### Impact of sorafenib on chemosensitivity

**Figure 4:**
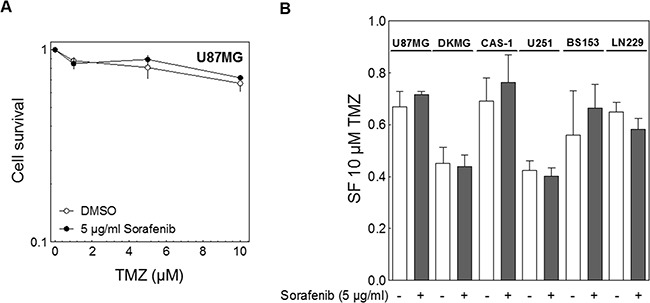
Effect of sorafenib on chemosensitivity Cells were treated with different concentrations of TMZ as indicated for 1-3 days according to their doubling time. The cells were also treated with 5 μg/ml sorafenib for the first 24 h. The medium was changed and cell survival was assessed by colony forming assay. **A.** Relative cell survival of U87MG cells. **B.** Relative survival fraction of all six GBM cell lines after 10 μM TMZ (SF10_TMZ_).

Besides irradiation TMZ is used in the standard treatment regime of GBM patients. Therefore we also analyzed the effect of sorafenib on TMZ-sensitivity using a clinically relevant dose range of 1 to 10 μM TMZ. Cells were treated with TMZ for 1-3 days according to their proliferation rate (Figure 4A; Supplementary Table S1). During this time sorafenib was present for the first 24 h. The addition of sorafenib did not influence the cell inactivation by TMZ and therefore had no impact on cellular chemosensitivity (Figure 4A, 4B).

Interestingly, the TMZ sensitivity varied quite strongly among the different cell lines although none of them expressed MGMT (Figure 1D). Furthermore, the TMZ sensitivity clearly did not correlate with cellular radiosensitivity (Supplementary Figure S2).

### Impact of sorafenib on combined treatment with irradiation and TMZ

**Figure 5:**
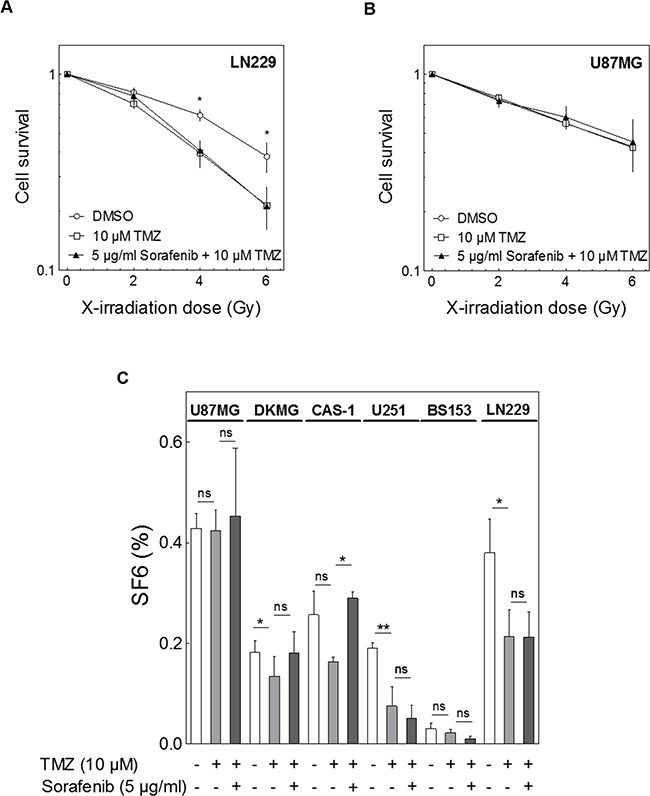
Impact of sorafenib on combined treatment with irradiation and TMZ Cells were treated with 10 μM TMZ with or without 5 μg/ml sorafenib 2 h before irradiation. Cellular survival was analyzed by colony formation. **A, B.** Relative cell survival of (A) LN229 and (B) U87MG cells. **C.** Relative surviving fraction at 6 Gy (SF6) with combined sorafenib and/or TMZ treatment as detected for all six cell lines.

Because GBM patients are generally treated with radiotherapy and adjuvant TMZ we also evaluated the effect of sorafenib on the combined treatment. In this experimental set up we also tested the effect of TMZ on cellular radiosensitivity alone and detected a radiosensitizing effect of TMZ in three of the six cell lines (Figure 5A, 5C; DKMG p_6Gy_=0.0252, U251 p_6Gy_=0.0041, LN229 p_6Gy_=0.0193) (Figure 5B, 5C). However, when sorafenib was added there was no further decrease in cell survival, but instead a significant increase was seen in Cas-1 cells (p_6Gy_=0.0303) (Figure 5C).

Taken together these results demonstrate that, although sorafenib inhibits proliferation of GBM cells and causes a modest cell inactivation as a single agent, it does not enhance cell inactivation by irradiation, TMZ or combined treatment.

## DISCUSSION

In this study, we analyzed the impact of sorafenib on cellular radiosensitivity, chemosensitivity (TMZ) and on the combined treatment of GBM cell lines. To consider potential variations which might affect the outcome of this study, we analyzed six individual GBM cell lines with differences in p53 and PTEN status, but all negative for MGMT expression (Supplementary Table S1).

Surprisingly, we observed no impact of sorafenib on cellular radiosensitivity in any of the cell lines. Instead, there was a trend towards radioresistance (Figure 3). This result is in contrast to the sorafenib-mediated cellular radiosensitization observed for other entities such as head and neck cancer, colorectal carcinoma, breast cancer and hepatocellular carcinoma [17–21]. Consequently, radiosensitization by sorafenib seems to be a frequent phenomenon in specific entities but not a general one.

Our results do not agree with data from former studies reporting an effect of sorafenib on the radiation response of GBM cell lines [22, 23]. However, these studies did not address radiosensitization in terms of clonogenicity. That radiosensitizing effects can in fact be observed using the clonogenic assay is demonstrated by the successful radiosensitization of three of the six cell lines by TMZ (Figure 5). This observation is in line with previous data demonstrating the radiosensitizing effect of TMZ [27].

Similar to the cellular radiosensitivity results we also detected no chemosensitization by sorafenib in combination with TMZ, but again a trend towards resistance (Figure 4). The resistance towards IR or TMZ&IR was unrelated to the ability of sorafenib to block MAPK-signaling (Supplementary Figure S3).

So far there are no other preclinical data available addressing this question. However, in combination with other drugs sorafenib was reported to enhance cytotoxicity in GBM cells but in these studies cellular survival was not analyzed in terms of clonogenicity [28–30]. Moreover, sorafenib might also have negative effects in combination with additional drugs, since it has been shown to reduce drug uptake [12, 31].

Although we demonstrate here, that sorafenib does not increase radiation- or TMZ-induced cell inactivation we observed a strong inhibition of proliferation which might translate into reduced tumor growth. But when sorafenib was removed after 24 h, proliferation restarted, resulting only in a minor delay of cell growth and a small reduction in clonogenicity for most of the cell lines (Figure 1C), which will likely have no strong influence on tumor control. However, sorafenib might improve tumor control in a clinical setting via additional mechanisms like targeting specifically tumor stem cells or influencing tumor angiogenesis [4, 32].

Sorafenib also had no positive effect on the double treatment with irradiation and TMZ. In fact, it instead made two of the six cell lines more resistant, which was significant for Cas-1 cells. These data are in line with recent clinical studies testing sorafenib in the first-line treatment in combination with TMZ after RCT (maintenance therapy) or in combination with TMZ and radiotherapy. These studies also do not support these treatment combinations because they did not seem to improve the efficacy of the treatment but caused increased side effects [33, 34].

Taken together, we have systematically demonstrated here for the first time that for GBM sorafenib induces only minor cell inactivation as a single treatment and has no benefit in respect to cell inactivation when combined with TMZ and/or irradiation. Moreover, sorafenib seems to cause resistance towards TMZ-based RCT as detected by increased cell survival in some cell lines. Therefore, our data do not support the use of sorafenib for GBM with the intention to increase cellular inactivation by RT or TMZ-based RCT.

## MATERIALS & METHODS

### Inhibitors and reagents

Small molecule inhibitor sorafenib (sorafenib tosylate, Nexavar^®^, Bayer HealthCare), alkylating agent temozolomide (10 μM, Sigma-Aldrich), solvent DMSO (Sigma-Aldrich).

### Cell culture

All the cell lines had already been used in the lab as reported earlier [26]. The GBM cell lines U87MG, CAS-1, U251, BS153 and LN229 were grown in DMEM (Sigma-Aldrich) supplemented with 10% FCS (Biochrom), 2 mM L-glutamine and 1 mM sodium pyruvate (Sigma-Aldrich). The GBM cell line DKMG was cultured in RPMI with 10% FCS, 2 mM L-glutamine, 1 mM sodium pyruvate. All cells were cultured at 37°C, 5% CO_2_ and 100% humidification and were identified by a short tandem repeat multiplex assay (Applied Biosystems). Gene sequencing and literature search revealed p53 wildtype expression in LN229, U87MG and DKMG cells and expression of mutant p53 in CAS-1, U251 and BS153 cells. Wildtype PTEN was detected only in LN229 cells (Supplementary Table S1). As for BS153 and DKMG cells the EGFRvIII-negative sublines were used [26].

### Cell proliferation

To analyse proliferation, 1×10^5^ cells were seeded. One day later, the cells were treated with sorafenib or DMSO (day 0) as indicated and the cell number was determined in parallel cultures by counting trypsinised cells using a Coulter counter (Beckman Coulter GmbH) every day henceforward.

### Irradiation

Cells were irradiated at room temperature with 200 kV X-rays (Gulmay RS225, Gulmay Medical Ltd., 15 mA, 0.8mm Be + 0.5 mm Cu filtering; dose rate of 1.2 Gy/min).

### Western blot

Proteins from whole cell extracts were detected by Western blot according to standard protocols. Primary antibodies: anti-ERK, anti-pERK (T202/Y204), anti-MEK1/2, anti-pMEK1/2 (S217/S221), anti-MGMT (Cell Signaling Technology) and anti-β-actin (Sigma-Aldrich). Secondary antibodies: anti-mouse and anti-rabbit (Li-COR Biosciences). The Odyssey^®^ CLx Infrared Imaging System (LI-COR) was utilized for signal detection and quantification.

### Colony forming assay (clonogenicity)

To analyse the capacity of the GBM cells for self-renewal (clonogenicity), the colony-forming assay was used. To this end, 200-350 cells (depending on the cell line) were seeded in triplicates 24 h prior to treatment with sorafenib (5 μg/ml), TMZ (10 μM) and/or irradiation. Twenty-four hours after treatment the medium was changed and TMZ was added to the corresponding samples for additional 1-2 days according to the doubling time of each cell line (Supplementary Table S1). The cells were grown until the colonies of all treatment arms had reached equal colony size (approximately 10-25 days; pre-plating conditions). Colony formation of U87MG, DKMG, CAS-1 and BS153 cells had to be promoted by replacing the medium in all colony assays by AmnioMax C-100 Basal Medium (Life Technologies) containing 10% FCS and C-100 supplement (Life Technologies) 24 h after irradiation. The cells were fixed with 70% ethanol, stained with crystal violet and colonies of more than 50 cells were counted manually. The surviving fraction of treated cells was normalized to the plating-efficiency of non-treated cells. For combination treatments involving radiation, the cells were irradiated 2 h after adding sorafenib and TMZ to minimise changes in the cell cycle distribution (verified by flow cytometry, data not shown). Sorafenib was removed 24 h later and TMZ was added for additional 1-2 days as mentioned above.

### Data evaluation

The experiments were repeated at least three times. The data were presented as mean values (±SEM). Prism software (GraphPad Prism 5) was used for analyzing and graphing the data. Student's t-tests were performed for the statistical analysis. P-values were calculated using unpaired two-sided tests (* p < 0.05, **p < 0.01).

## SUPPLEMENTARY FIGURES AND TABLE


