# Supplementary material for: Sorafenib inhibits cell growth but fails to enhance radio- and chemosensitivity of glioblastoma cell lines
Source: Oncotarget. 2016 Aug 17;7(38):61988–95. doi: 10.18632/oncotarget.11328 (PMC5308705; doi:10.18632/oncotarget.11328)
Supplement: Supplementary file 1 [file oncotarget-07-61988-s001.pdf]

Sorafenib inhibits cell growth but fails to enhance radio- and chemosensitivity of glioblastoma cell lines

SUPPLEMENTARY FIGURES AND TABLE

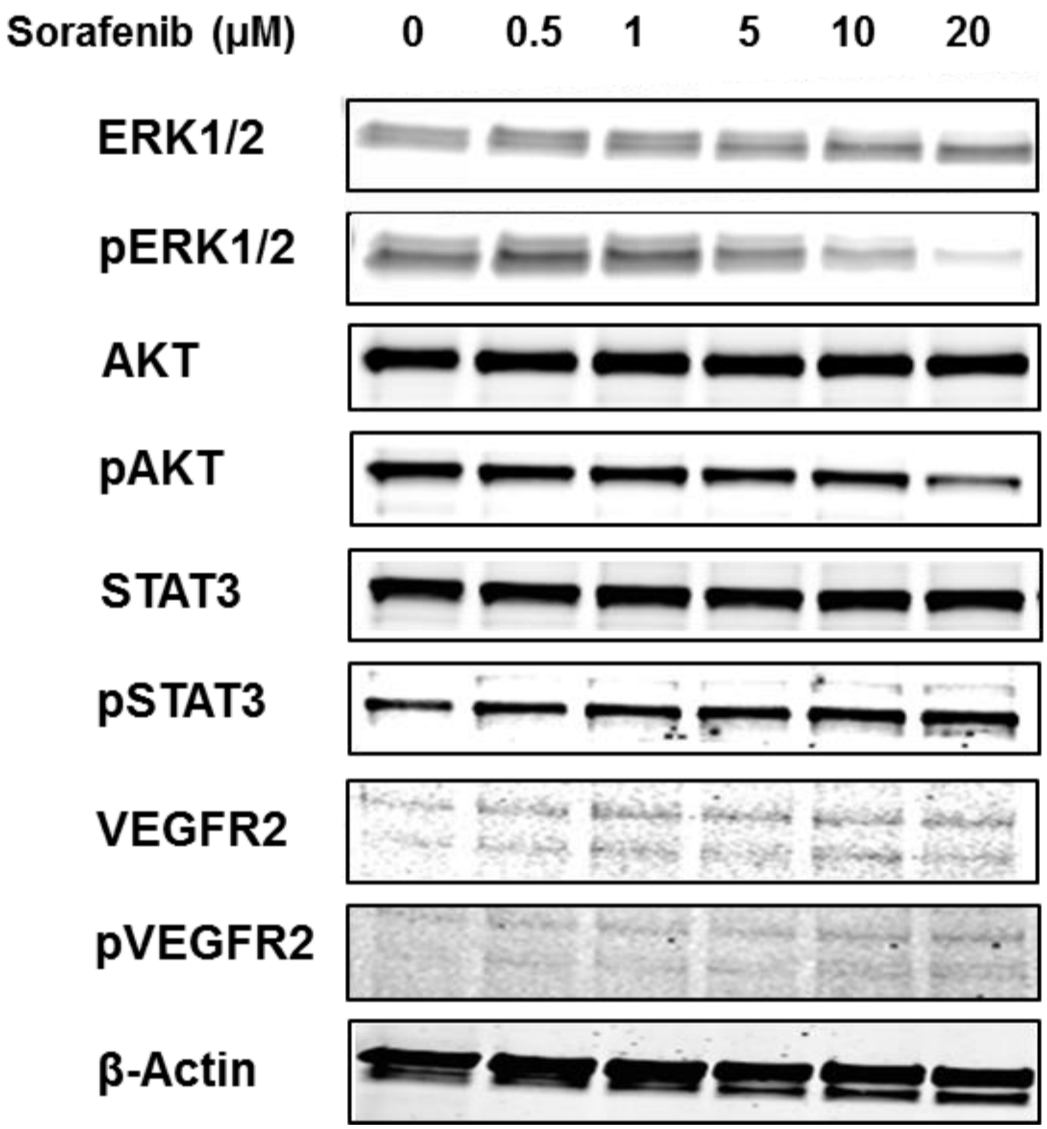

Supplementary Figure S1: Effect of sorafenib on signal transduction. The phosphorylation of potential sorafenib targets was analyzed in DKMG cells using phosphospecific antibodies in Western blot experiments as indicated (pERK, T202/Y204; pAKT, T308; pSTAT, Y1059; pVEGFR2, Y705). The total proteins were detected as a control. Prior to lysis cells were treated with increasing concentrations of sorafenib for 2 h.

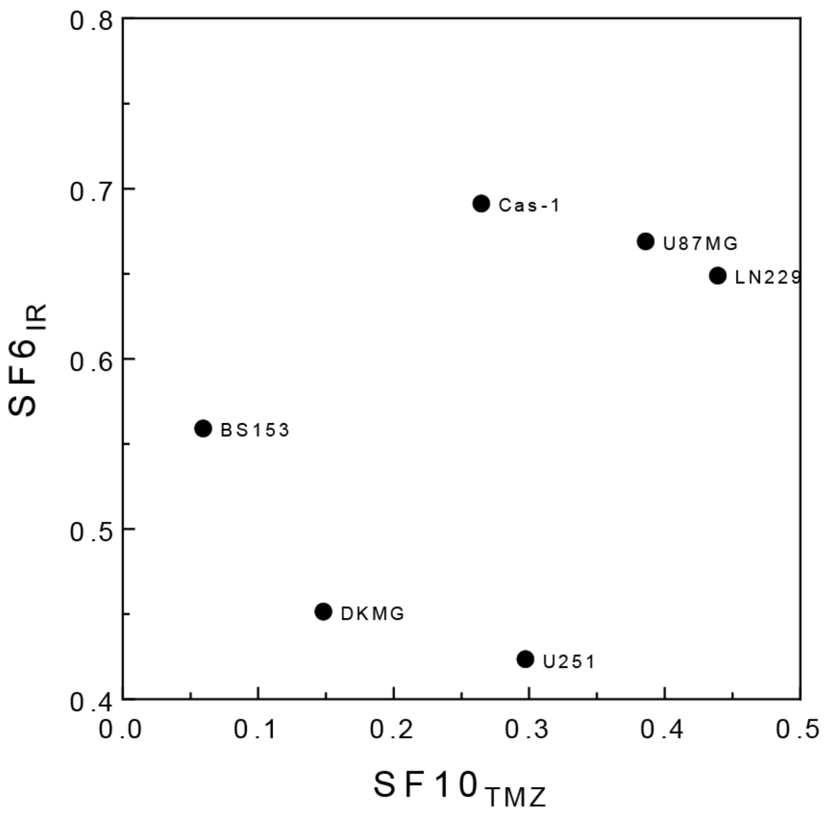

Supplementary Figure S2: SF6 vs. TMZ-sensitivity. No correlation of cellular radiosensitivity ($SF6_{Gy}$) and cellular chemosensitivity ($SF10_{TMZ}$) in GBM cell lines. Data were taken from Fig.3B ($SF6_{Gy}$) and Fig.4B ($SF10_{TMZ}$).

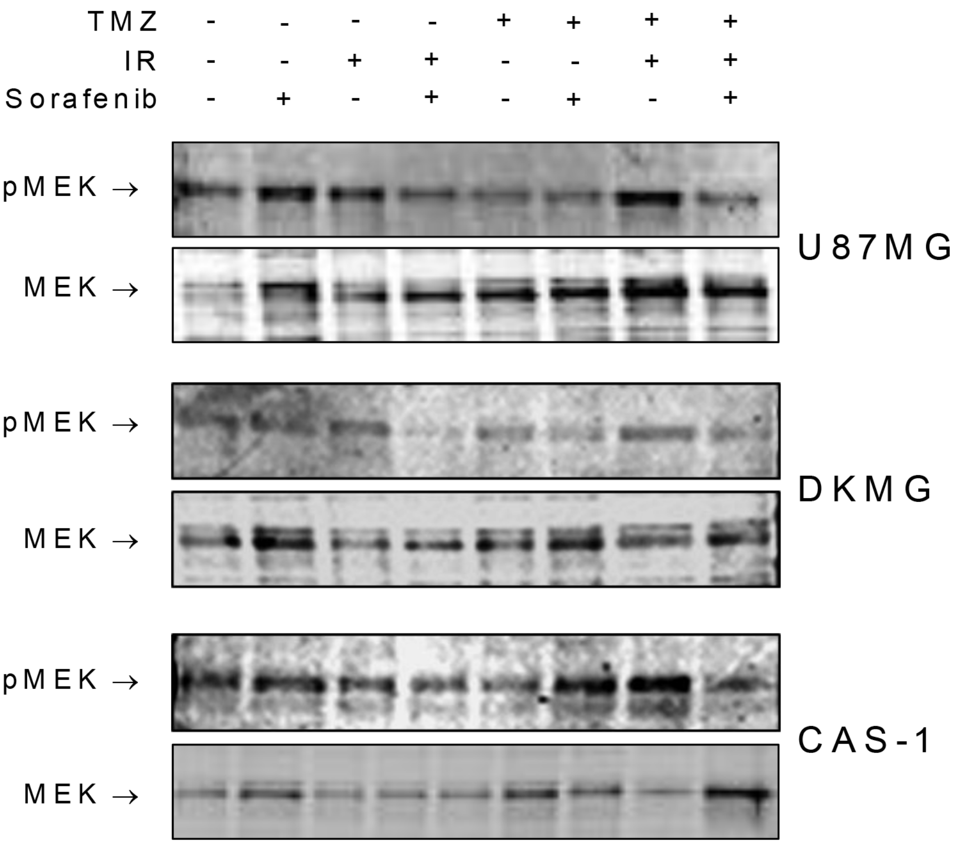

Supplementary Figure S3: Effect of sorafenib on MAPK signaling after IR and TMZ treatment. In U87MG, DKMG and Cas-1 cells the phosphorylation of MEK1/2 (S217/S221) was analyzed by Western blot. Total MEK was detected as a control. Prior to lysis cells were treated with 5 µg/ml sorafenib and/or 10 µM TMZ 2 h before 6 Gy of irradiation as indicated.

Supplementary Table S1: Characterization of GBM cells

Cell line	p53 status	PTEN status	EGFRvIII	doubling time, d
U87MG	wt[1]	mut[2]	-[3]	1*
DKMG	wt[3]	mut (T167A)[3]	-[3]	2*
Cas-1	mut (R248W)[^]	not expressed[^]	-[3]	2-3*
U251	mut (R273H)[4]	mut[2]	-[3]	1*
BS153	mut (R248Q)[3]	mut(C136Y)[3]	-[3]	3*
LN229	wt[5]	wt[6]	-[3]	<1*

[^]own analysis; sequencing of the PTEN gene (exons 1-9) and TP53 (exons 5-8)
*own data (not shown); values were extracted from the cell proliferation analysis (see *Materials & Methods*)

SUPPLEMENTARY DATA

1. Cerrato JA, Yung WK, Liu TJ. Introduction of mutant p53 into a wild-type p53-expressing glioma cell line confers sensitivity to Ad-p53-induced apoptosis. Neuro Oncol. 2001; 3: 113-22.
2. Pore N, Liu S, Haas-Kogan DA, O'Rourke DM, Maity A. PTEN mutation and epidermal growth factor receptor activation regulate vascular endothelial growth factor (VEGF) mRNA expression in human glioblastoma cells by transactivating the proximal VEGF promoter. Cancer Res. 2003; 63: 236-41.
3. Struve N, Riedel M, Schulte A, Rieckmann T, Grob TJ, Gal A, Rothkamm K, Lamszus K, Petersen C, Dikomey E, Kriegs M. EGFRvIII does not affect radiosensitivity with or without gefitinib treatment in glioblastoma cells. Oncotarget. 2015; 6: 33867-77. doi: 10.18632/oncotarget.5293.
4. Brazdova M, Quante T, Togel L, Walter K, Loscher C, Tichy V, Cincarova L, Deppert W, Tolstonog GV. Modulation of gene expression in U251 glioblastoma cells by binding of mutant p53 R273H to intronic and intergenic sequences. Nucleic Acids Res. 2009; 37: 1486-500. doi: 10.1093/nar/gkn1085.
5. Trepel M, Groscurth P, Malipiero U, Gulbins E, Dichgans J, Weller M. Chemosensitivity of human malignant glioma: modulation by p53 gene transfer. J Neurooncol. 1998; 39: 19-32.
6. Wick W, Furnari FB, Naumann U, Cavenee WK, Weller M. PTEN gene transfer in human malignant glioma: sensitization to irradiation and CD95L-induced apoptosis. Oncogene. 1999; 18: 3936-43. doi: 10.1038/sj.onc.1202774.
